# Antiproliferative and apoptotic activity of gemcitabine-lauric acid conjugate on human bladder cancer cells

**DOI:** 10.22038/IJBMS.2022.61118.13528

**Published:** 2022-04

**Authors:** Hongxia Wang, Zhiyu Shao, Zhiwen Xu, Binghao Ye, Ming Li, Qiaoqiao Zheng, Xingyuan Ma, Ping Shi

**Affiliations:** 1 State Key Laboratory of Bioreactor Engineering, East China University of Science and Technology, Shanghai 200237, China; 2 College of Chemistry, Chemical Engineering and Biotechnology, Donghua University, Shanghai 201620, China

**Keywords:** Apoptosis, Cell cycle checkpoints, Gemcitabine, SLC29A1 protein, Urinary bladder neoplasms

## Abstract

**Objective(s):**

Gemcitabine is a first-line drug for the treatment of bladder cancer. One of the most important mechanisms of gemcitabine resistance is the low expression of cellular membrane transporter hENT1. Various derivatives containing fatty acid side chains have been developed in order to facilitate gemcitabine uptake and prolong its retention in cells, such as CP-4126. In this study, the anti-tumor effect and mechanism of a new derivative of gemcitabine named SZY-200 on bladder cancer cells were investigated. SZY-200 was assembled from the gemcitabine-lauric acid conjugate.

**Materials and Methods:**

Antiproliferative activities of SZY-200 and lauric acid were evaluated using CCK-8 assay and clonogenic survival assay. The hENT1 inhibitor NBMPR was employed to determine the role of hENT1 in the apoptotic activity of GEM, CP-4126, and SZY-200. RT-qPCR, flow cytometry, fluorescence microscope, western blotting, and wound healing assay were used to study the mechanisms of SZY-200. The target genes were predicted using the BATMAN-TCM database.

**Results:**

Our data showed that SZY-200 could inhibit the proliferation of bladder cancer cells by inducing cell cycle arrest and apoptosis. The inhibitory effects were comparable to gemcitabine and CP-4126. SZY-200 does not rely on hENT1 to help it enter bladder cancer cells. Also, we found that lauric acid could inhibit the proliferation of bladder cancer cells. SZY-200 could down-regulate the expressions of PPARG and PTGS2 which were related to the occurrence and development of bladder cancer.

**Conclusion:**

SZY-200 has the same or more advantages as CP-4126 and could be an ideal candidate drug for further *in vivo* investigation.

## Introduction

Bladder cancer is the 10th most commonly diagnosed cancer worldwide, with approximately 573,000 new cases and 213,000 deaths in 2020 (1). Gemcitabine (GEM) is currently the first-line option for treating bladder cancer and is widely used for bladder perfusion therapy in patients with non-muscle invasive bladder cancer (NMIBC). Intravesical GEM seemed to be a valid and safe alternative after 3 years of follow-up for patients who were unwilling to undergo radical cystectomy (RC) or failed bacillus Calmette-Guérin (BCG) (2). GEM is the most effective therapy considering both recurrence and progression outcomes (3). In addition, the combination chemotherapy of GEM and cisplatin (GC) exhibited similar efficacy and fewer adverse complication for patients with MIBC than the standard methotrexate plus vinblastine, doxorubicin, and cisplatin (MVAC) therapy and high-dose MVAC (4, 5). However, drug resistance and toxicity severely limit utilization of GEM in clinics. GEM is a highly hydrophilic pyrimidine nucleoside analog. Human equilibrative nucleoside transporter 1 (hENT1) is the primary facilitative transporter of GEM (6), which is localized to the plasma and mitochondrial membranes in cells (7). High levels of hENT1 were correlated with high GEM concentration in cancer cells and increased drug efficacy (8, 9). Previous studies indicated that high expression of hENT1 in tumor cells was associated with prolonged survival in patients with metastatic bladder cancer treated with gemcitabine-cisplatin-based combination chemotherapy (10). High hENT1 expression was also associated with longer overall survival (OS) among patients with pancreatic cancer treated with both palliative and adjuvant GEM (11, 12) and vice versa (13, 14). The activation of GEM by deoxycytidine kinase (dCK) to form active diphosphate and triphosphate forms (dFdCDP and dFdCTP, respectively) is the rate-limiting step (15). Furthermore, the majority (~ 90%) of intracellular GEM is inactivated by deamination by deoxycytidine deaminase (dCDA) to 2’, 2’-difluorodeoxyuridine (dFdU) and subsequently degraded and excreted out of cells (16). Additionally, oral administration of GEM was terminated because of high conversion of GEM and accumulation of dFdU, which most likely resulted in severe liver toxicity (17). Therefore, it is urgent to further modify and design novel GEM derivatives.

CP-4126 is a GEM derivative assembled from GEM-elaidic acid (EA) conjugate, which has a broad-spectrum anti-tumor activity in a wide range of human tumor models *in vivo *(18, 19). Importantly, some studies have shown that CP-4126 is membrane transporter independent. CP-4126 is not the substrate for dCDA and can inhibit deamination of GEM (19-21). However, EA, another hydrolyzed product of CP-4126 was reported to enhance the metastasis of colon cancer by increasing the stemness of cancer cells and epithelial-mesenchymal transition and inducing drug resistance (22). In contrast, lauric acid (LA) is associated with certain health benefits of coconut oil intake and some studies highlighted its anti-tumor effects. LA contributes the least to fat accumulation among all fatty acids (23). A study (24) reported that a diet regimen of ketogenic breakfast along with supplementation with two doses of LA-rich medium-chain triglycerides at breakfast and lunchtimes could be a potential prophylactic strategy and adjuvant therapy to combat COVID-19 infections. LA could stimulate ketone body production in the KT-5 astrocyte cells, which improved brain health by providing fuel to neighboring neurons (25). Moreover, LA has anti-proliferative and pro-apoptotic efficacy in colon, breast, and endometrial cancer cells (26). Therefore, researchers designed a new active derivative of GEM by using LA to replace the EA in CP-4126 and named it SZY-200.

The present study aims to investigate the potential anti-tumor effect of SZY-200 on bladder cancer cells and to further understand the underlying molecular mechanism in an effort to shed light on the potential development of SZY-200 in the treatment of bladder cancer, and even other types of cancers.

## Materials and Methods

***Chemicals***

**Figure 1:**
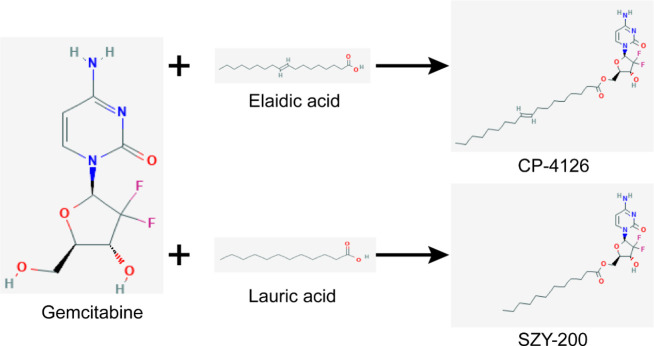
Design strategies and chemical structures of GEM derivatives CP-4126 and SZY-200

GEM was bought from Shanghai Macklin Biochemical Company. CP-4126 and SZY-200 were synthesized by Shao Zhiyu (Donghua University, Shanghai, China). They were dissolved in DMSO (Solarbio Life Science, Beijing, China) to a final concentration of 8 mM and stored at 4 ^°^C for *in vitro* assay. LA and EA were purchased from Aladdin (Shanghai, China) and Alfa Aesar (Ward Hill, MA), respectively. They were dissolved in absolute ethanol to a final concentration of 60 mM and stored at room temperature. The chemical structures of these drugs were shown in Figure 1. Nitrobenzylthioinosine (NBMPR) was purchased from Topscience (Shanghai, China) and dissolved in DMSO to a final concentration of 100 mM, and stored at -20 ^°^C. Propidium iodide (PI) was bought from Sangon Biotech (Shanghai, China). RNase A was purchased from Solarbio Life Science (Beijing, China).

***Cell culture***

The human normal uroepithelium cell line SV-HUC-1 was purchased from the American Type Culture Collection (ATCC; Manassas, VA, USA) and maintained in DMEM/F12 medium (Gibco/Invitrogen, Camarillo, CA, USA). Human bladder cancer cell lines (T24, UM-UC-3, and 5637), human normal hepatocyte cell line QSG-7701, human lung cancer cell line A549, and colon cancer cell line HCT116 were acquired from the Chinese Academy of Sciences (Shanghai, China) and maintained in RPMI-1640 medium (Gibco) or DMEM (Gibco) and supplemented with 1% penicillin G sodium/streptomycin sulfate and 10% fetal bovine serum (FBS, PAN-Biotech, Aidenbach, Germany) in a humidified atmosphere composed of 5% CO_2_ and 95% air at 37 ^°^C.

***Cell viability assay***

The cells in the logarithmic phase were seeded in 96-well plates and incubated at 37 ^°^C to allow cell attachment, then treated with GEM, CP-4126 or SZY-200 (0, 1, 5, 25, and 50 nM), LA or EA (150 and 300 μM) for 48 h. The nucleoside transport carrier inhibitor NBMPR (100 μM) was added to the cells for 1 hr and removed before adding the drugs. The CCK-8 kit was used to measure cell viability according to the manufacturer’s instructions (APExBIO, Houston, USA). In brief, after adding 10 μl CCK-8 to each well and incubating for 1 hr at 37 ^°^C, absorbance at 450 nm was measured by the microplate reader (Bio-Rad, Hercules, USA). The software GraphPad Prism 8.4 (GraphPad Software, Inc.) was used to perform statistical analysis of cell viability results. The experiment was performed in triplicate and repeated at least three times.

***Cell colony-forming assay***

UM-UC-3 cells were seeded in 6-well plates (1000 cells/2 ml medium/well) and incubated at 37 ^°^C for 24 hr, then treated with LA and EA (150 and 300 μM) for 7-10 days for colony growth. After the medium was removed, the cells were fixed with 4% paraformaldehyde for 20 min at room temperature and stained with 0.1% crystal violet for 30 min, and the plate was imaged and colonies were counted.

***Quantitative real-time PCR (qRT-PCR)***

Total RNA in cells was extracted using the Trizol reagent (Yeasen Biotech, Shanghai, China) and reverse-transcribed using the Hifair® II 1^st^ Strand cDNA Synthesis kit (Yeasen Biotech) according to the manufacturer’s instructions. qRT-PCR was performed on the C1000 Thermal Cycle system (Bio-Rad, Hercules, USA) using Hieff UNICON® qPCR SYBR Green Master Mix (Yeasen Biotech). PCR conditions were as follows: 1 cycle of 95 ^°^C for 30 sec followed by 40 cycles of 95 ^°^C for 5 sec and 60 ^°^C for 30 sec. The genes primer sequences were listed in Table 1. Transcript quantities were compared by the relative Ct method. 18S ribosomal RNA was used as the reference gene to normalize target genes’ expression. We evaluated PCR efficiency by the serial dilution series of targets (data not shown). The value in relation to the control sample was given by 2^-ΔΔCt^. The experiment was performed in triplicate and repeated at least three times.

***Cell cycle analysis***

After incubating with drugs (12.5 nM) in 6-well plates for 24 hr, the cells were harvested, centrifuged, and washed with cold PBS twice. Then fixed in 1 ml ice-cold ethanol (70%) at -20 ^°^C overnight. After that, the cells were washed again with cold PBS and resuspended in cold PI solution (50 μg/ml) containing RNase A (0.1 mg/ml) in PBS (pH 7.4) for 30 min at 37 ^°^C in the dark. The cell cycle was measured by a CytoFLEX flow cytometer (Beckman Coulter, Brea, USA). The experiment was performed in triplicate and repeated at least three times.

***Apoptosis analysis***

The apoptosis of cells was observed using the Hoechst 33258 assay kit (Dojindo Laboratories, Tokyo, Japan). The cells were seeded in 6-well plates and treated with drugs (12.5 and 25 nM). After 48 hr, the attached cells were washed with PBS and fixed in freshly prepared 4% paraformaldehyde for 20 min at room temperature, then washed with PBS and incubated with Hoechst 33258 staining solution for 10 min at 37 ^°^C in the dark. After that, cells were washed with PBS and Antifade Mounting Medium was added, then observed under a fluorescence microscope (Olympus BX51, Tokyo, Japan).

The proportion of apoptotic cells was quantified using the Annexin V-FITC/PI Kit by flow cytometry. After incubation with drugs (25 nM) in 6-well plates for 48 hr, the cells were harvested and centrifuged, washed with PBS, stained with 5 μl Annexin V-FITC and 10 μl propidium iodide in the dark at room temperature for 15 min according to the manufacturer’s protocol (Yeasen Biotech) and then determined with a CytoFLEX flow cytometer (Beckman Coulter, Brea, USA). Data analysis was performed with FlowJo software. The experiment was performed in triplicate and repeated at least three times.

***Western blotting analysis***

The cells were seeded in 6-well plates and treated with different drugs (12.5 and 25 nM). After 24 or 48 hr, the cells were washed with ice-cold PBS and lysed on ice for 30 min in lysis buffer containing 400 mM NaCl, 1.5 mM MgCl_2_, 25 mM HEPES (pH 7.7), 0.5% Triton X-100, 2 mM EDTA, 10 mM DTT, 0.1 mM PMSF, 20 mM β-GP, and 1 μM Na_3_VO_4_. The lysate was centrifuged at 12,000 × g for 15 min, the supernatant was collected, and protein concentration was determined by Bradford assay. 40 μg proteins from each sample were subjected to electrophoresis on 12% SDS-PAGE. The separated proteins were transferred onto a PVDF membrane. After being blocked with 5% skimmed milk in PBST at room temperature for 2 hr, the PVDF membrane was incubated with primary antibodies for GAPDH (KC-5G5, Aksomics, Shanghai, China), Bax (AF0120, Affinity biosciences OH, USA), Bcl-2 (AF6139, Affinity biosciences OH, USA) and Caspase-3 (14220, Cell Signaling Technology, USA) at 4 ^°^C overnight, respectively. The membranes were washed three times (10 min per wash) and then incubated with a goat anti-rabbit IgG HRP secondary antibody (111-036-003, Jackson ImmunoResearch Laboratories, USA) at room temperature for approximately 2 hr and washed again. The specific protein signals were visualized using ECL Protein Imprint Detection Kit (EpiZyme, Shanghai, China), followed by exposure with the Tanon 6200 Imaging System (Tanon Science & Technology Co., Ltd, Shanghai, China).

***Wound healing assay***

The cells were seeded in 6-well plates and cultured until 100% confluence. A scratch was created using a 10 μl pipette tip and cells were washed with PBS. Serum-free medium containing different drugs was added to allow cells to move into the gap. At 0, 20, 28, and 40 hr wound closure images were captured in the same pre-marked field under magnification. Cell motility was calculated as the ratio of the difference between the initial scratch area and the final scratch area of each sample to the initial scratch area. The experiment was performed in triplicate and repeated at least three times.

***Explore target genes of GEM and LA***

The target genes of GEM and LA were predicted using BATMAN-TCM database(27) (http://bionet.ncpsb.org.cn/batman-tcm/index.php) (with score cutoff=20 and adjusted *P*-value=0.05).

***Statistical analysis***

All experiments were performed in triplicate and repeated at least three times. The data were expressed as the mean± SEM using GraphPad Prism 8.4 statistical software. Statistical analysis was performed using one-way ANOVA followed by Dunnett’s multiple comparisons test. A *P*-value less than 0.05 was considered statistically significant.

## Results

***SZY-200 and LA suppress proliferation of bladder cancer cells***

**Figure 2:**
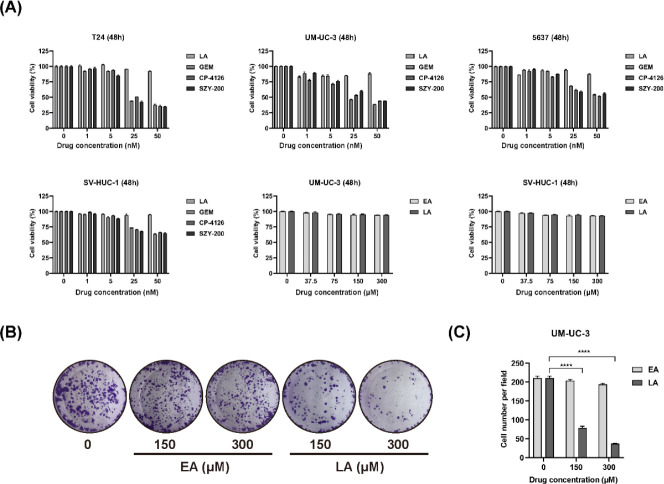
Antiproliferative activities of SZY-200 and LA against tumor cell lines with CP-4126, GEM, and EA as positive control. (A) Cell viability after treatment for 48 hr. (B) Colony formation abilities of UM-UC-3 cells were determined after treatment with EA or LA. (C) Statistical analysis of B. Values are expressed as the mean±SEM from three independent experiments. ****P<0.0001 vs 0 μM

Since GEM is a standard chemotherapeutic agent for the treatment of various cancers, we wondered whether SZY-200 shows a similar effect. Firstly, we evaluated the effects of SZY-200 and LA on the proliferation of various cells *in vitro* using the CCK-8 assay. GEM and CP-4126 were used as positive controls. We found that SZY-200 could significantly inhibit the proliferation of a variety of cancer cells in a dose-dependent manner and has a certain selectivity to the normal uroepithelium cell line SV-HUC-1 and human normal liver cell line QSG-7701 (Figures 2A and S1). Moreover, SZY-200, GEM, and CP-4126 showed similar cytotoxic effects at the same dose and the human bladder cancer cells were more sensitive to them. While LA did not affect the proliferation of any tested cells at the same dose (Figures 2A and S1). We also compared the effects of LA with EA on cell proliferation. The data showed that LA and EA had no significant growth inhibitory effects on UM-UC-3 and SV-HUC-1 cells (Figure 2A). However, the cell colony-forming assay showed that LA could significantly inhibit the proliferation of UM-UC-3 cells in a dose-dependent manner. While EA had no inhibitory effect on the cells (Figures 2B, C).

***SZY-200 is membrane transport system independent***

**Figure 3:**
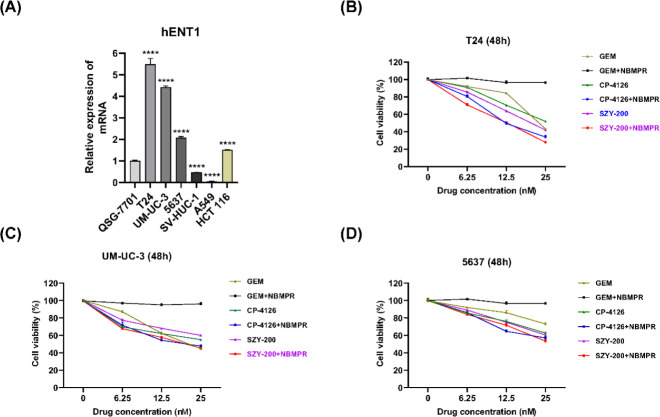
Expressions of hENT1 at the transcriptional level in different cells and the effects of the nucleoside transport inhibitor NBMPR on the cytotoxic activities of SZY-200, GEM, and CP-4126 in bladder cancer cells. (A) Expression of hENT1 in different cells, ****P<0.0001 vs the normal cells QSG-7701. Cell viability was measured at 48 hr after T24 cells (B) UM-UC-3 cells (C), and 5637 cells (D) were treated with SZY-200, GEM, and CP-4126 (6.25, 12.5, and 25 nM) with or without 100 μM NBMPR. Data were mean±SEM (n=3)

The differential expressions of nucleoside transport hENT1 mRNA in different cells were analyzed by qRT-PCR. Compared with other types of cell lines used in our study, the three bladder cancer cells exhibited high levels of hENT1 mRNA (Figure 3A). The data showed that the expression levels were basically consistent with the sensitivities of the cells to the three drugs (Figures 3A, 2A, and S1). Subsequently, to study the role of hENT1 for these three drugs, differences in sensitivity to the drugs in the presence of hENT1 inhibitor NBMPR were determined. NBMPR decreased sensitivity to GEM in bladder cancer cells, while sensitivities to SZY-200 and CP-4126 were increased (Figures 3B-3D). Taken together, different from GEM, SZY-200 and CP-4126 were independent of the membrane transport system. That is, they do not rely on hENT1 to help them enter bladder cancer cells.

***SZY-200 could arrest the cell cycle, induce apoptosis, and significantly inhibit bladder cancer cells migration***

**Figure 4:**
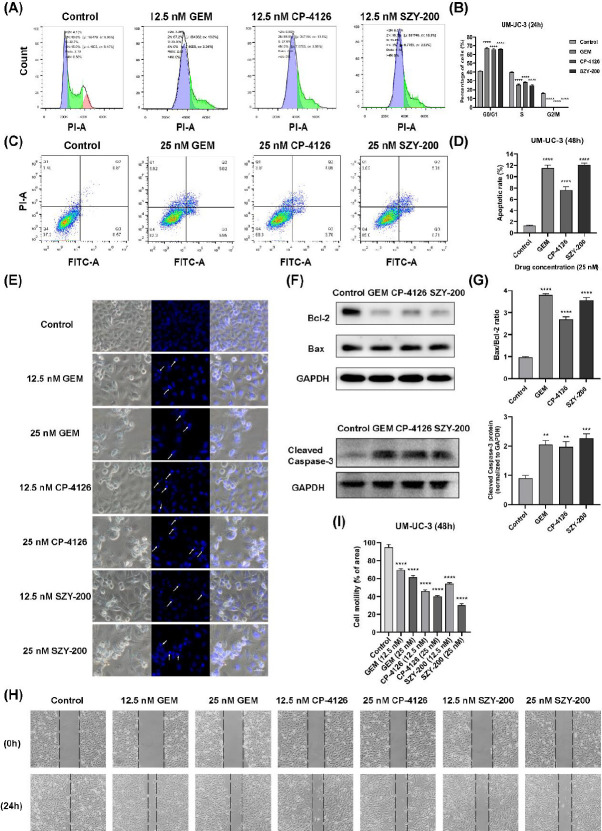
Effects of SZY-200 on cell cycle progression and apoptosis rate in UM-UC-3 cells and scratch tests. (A) After incubation, the cell cycle of UM-UC-3 cells was analyzed by flow cytometry. (B) Statistical analysis of the percentage of cells at the G0/G1, S, and G2/M phases of the cell cycle. (C) Apoptotic analysis was performed by flow cytometry using Annexin V-FITC/PI double staining method. (D) Statistical analysis of the percentages of apoptotic cells. (E) Apoptosis in UM-UC-3 cells was observed by a fluorescence microscope using Hoechst 33258 staining (Scale bar=50 µm). (F) Cell apoptosis-related protein expression in UM-UC-3 cells was detected by western blotting. (G) Histograms represent the ratio of Bax/Bcl-2 and cleaved caspase-3 protein expression levels. **P<0.01, ***P<0.001, and ****P<0.0001 vs control. (H) Representative wound-healing assay pictures for UM-UC-3 cells treated with three drugs (12.5 and 25 nM) are shown (vertical lines indicate wound edges); (400×, original magnification). (I) Histograms represent quantitative analyses of cell migration. Values are expressed as the mean±SEM from three independent experiments. ****P<0.0001 vs control

Flow cytometric analysis was performed to determine whether SZY-200 affected the cell cycle distribution in bladder cancer cells. As shown in Figures 4A and 4B, similar to GEM and CP-4126, SZY-200 significantly induced G1-phase cell cycle arrest in UM-UC-3 cells. In T24 and 5637 cells, SZY-200 also was similar to GEM and CP-4126 and caused the cell cycle arrest in the G2 phase (Figures S2A and S2B).

Then, we quantified the SZY-200-induced apoptosis of cells by flow cytometry. The percentage of total apoptotic cells was significantly increased in UM-UC-3 (Figures 4C and 4D), T24, and 5637 (Figure S2C and S2D) cells after being treated by SZY-200, GEM, and CP-4126. Meanwhile, Hoechst 33258 staining showed changes in cellular morphology in UM-UC-3, T24, and 5637 cells after being treated by SZY-200, GEM, and CP-4126 (Figures 4E, S2E). The untreated control cells were regular in shape, with abundant cytoplasm, and also well attached to the membrane, the nuclei were round and stained homogeneously. On the contrary, the cells treated with three drugs were irregular in shape, the cell boundary was not smooth and the cells were more loosely attached to the membrane, there was lysis of some cells and showed evidence of blebbing. Moreover, apoptotic bladder cancer cells showed condensed and marked fragmented nuclei (white arrows) in a dose-dependent manner. Western blot analysis showed that the ratio of apoptosis-related proteins Bax and Bcl-2 and expressions of cleaved Caspase-3 were increased in UM-UC-3 cells after being treated by SZY-200, CP-4126, and GEM (Figures 4F, G). Similar data were obtained in T24 cells except for no change in the ratio of Bax and Bcl-2 after being treated by GEM (Figures 2F, G). Taken together, these data suggest that SZY-200 induced apoptosis of bladder cancer cells.

Subsequently, cell motility was assessed by light microscopy. As shown in Figures 4H and I, three drugs could significantly inhibit the migration of UM-UC-3 cells in a dose-dependent manner. Similar data were observed in both T24 and 5637 cells (Figures 3A, B).

***SZY-200 down-regulates the expression of peroxisome proliferator-activated receptor γ (PPARG), cyclooxygenase‐2 (COX‐2, also known as PTGS2) in bladder cancer cells***

**Figure 5:**
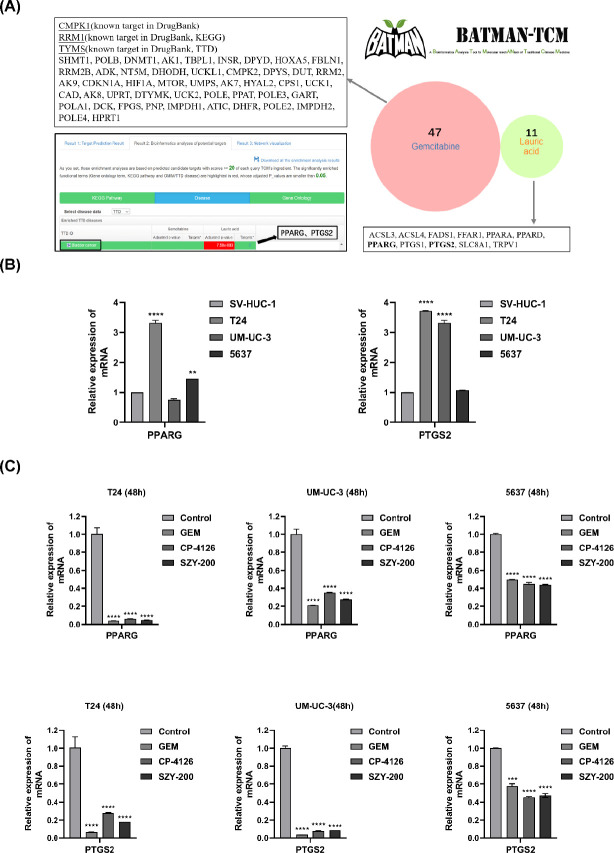
Prediction and analysis of target genes of GEM and LA. (A) Target genes from the BATMAN-TCM database and disease enrichment analysis from the TTD database. (B) Differential expressions of PPARG and PTGS2 in different cells. **P<0.01 and ****P<0.0001 vs SV-HUC-1. (C) Effects of three drugs on the expressions of PPARG and PTGS2 in bladder cancer cells. Values are expressed as the mean±SEM from three independent experiments. ***P<0.001, ****P<0.0001 vs control

Finally, the BATMAN-TCM database was used to predict the target genes of GEM and LA. As shown in Figure 5A, there are 47 target genes for GEM, 11 for LA, and no intersection between them. Disease enrichment analysis from the TTD database showed that the target genes of LA, PPARG, and PTGS2 were related to the occurrence and development of bladder cancer (as shown in the black box). Moreover, qRT-PCR analysis showed that the mRNA expression of PPARG and PTGS2 was up-regulated in two bladder cancer cell lines in comparison with the normal cells SV-HUC-1 (Figure 5B). Importantly, SZY-200, CP-4126, and GEM could down-regulate the expression of the two genes in bladder cancer cells (Figure 5C).

## Discussion

T24 and UM-UC-3 are human muscle-invasive urothelial bladder-cancer cell lines, and 5637 is a human superficial urothelial bladder-cancer cell line (one non-muscle-invasive cell line). In order to determine whether the anti-tumor activities and mechanisms of SZY-200 on different bladder-cancer cell lines are the same, we used these three different human bladder cancer cell lines. Our data showed that T24 is the most sensitive to SZY-200, UM-UC-3 is the next, and 5637 is the least sensitive (Figure 2A).

Previous studies have shown that CP-4126 was well tolerated with a comparable toxicity profile to GEM in patients with some advanced solid tumors (28). In this study, SZY-200 and CP-4126 also showed similar cytotoxic effects in bladder cancer cells compared with GEM. The similar effect of SZY-200, CP-4126, and GEM demonstrated an effective conversion of the derivatives to GEM. CP-4126 was reported to traverse cell membranes by passive diffusion, followed by intracellular conversion to GEM by esterases in plasma and within tumor cells to be activated, and it is also dependent on dCK for phosphorylation (29). In addition, CP-4126 is mainly localized in the membrane and cytosolic fraction, which leads to long retention inside the cell (21). Compared with CP-4126, SZY-200 is assembled with LA not EA. Liu *et al*. (30) once synthesized a drug (LA-Ara) based on the conjugation of cytarabine (Ara-C) with LA. LA increased the lipophilicity of Ara-C and protected its NH_2_ group from the enzymatic attachment, thus markedly prolonging its plasma half-life. Furthermore, LA-Ara effectively improved anti-tumor activity compared with Ara-C. Importantly, LA-Ara could obviously decrease the incidence of toxic effects of Ara-C and is suitable for oral administration. From our data, clonogenic survival assay showed that LA could significantly inhibit the proliferation of bladder cancer cells in a dose-dependent manner, while EA has no inhibitory effect (Figures 2B, C). Therefore, SZY-200 has the same or better anti-tumor activity than CP-4126.

Several previous studies highlighted a significant correlation between the levels of hENT1 expression and the IC_50_ values for GEM in cancer cells (31, 32). Research (33) demonstrated that patients with high hENT1 expression had a significantly higher median survival compared with patients with low hENT1 expression for those who received adjuvant GEM after undergoing surgical resection for pancreatic cancer. Galmarini *et al*. (20) found that the inhibition of hENT1 conferred resistance to GEM. In our study, the levels of hENT1 mRNA were found to basically be consistent with the sensitivities of the cells to GEM, CP-4126, and SZY-200 (Figure 3A). The hENT1 inhibitor, NBMPR, decreased sensitivity to GEM in bladder cancer cells and increased sensitivities to SZY-200 and CP-4126 (Figures 3B-D). Similarly, Bergman *et al*. (19) reported that NBMPR and another nucleoside transport inhibitor dipyridamole decreased sensitivities to GEM 55~273-fold, while sensitivities to CP-4126 were only decreased 0~1.8-fold in THX human malignant melanoma cells and MOLT4 human T-cell leukemia cells. These studies revealed a nucleoside transporter independent transport over the cell membranes of CP-4126 and SZY-200. Moreover, hENT1 could operate as uptake as well as export transporter of GEM, the activity of SZY-200 and CP-4126 could be improved by cellular accumulation and prolonged retention of GEM inside the cell because of the inactivation of hENT1 (21). We, therefore, believe that SZY-200 could be more effective than GEM for bladder cancer patients with low hENT1 expression. For another, GEM was reported to inhibit the proliferation of bladder cancer cells by inducing cell cycle arrest and apoptosis (34, 35). From our data, SZY-200 and CP-4126 exhibited similar effects, which suggests that these GEM derivatives will release GEM in tumor cells to play their anticancer role. Interestingly, these drugs induced cell cycle arrest at G1-phase in UM-UC-3 cells, but in G2-phase in T24 and 5637 cells (Figures 4A, 4B, S2A, and S2B). Several previous studies also showed similar results (36-38). The underlying mechanism of this difference needs to be further explored in the future. Regarding the apoptosis induced by these drugs, our current data showed that they at least activate the internal apoptotic pathway (Figures 4C-G, S2C-G). Whether they activate the external pathway of apoptosis remains to be further studied.

PPARG and PTGS2 are target genes of LA which we predicted by the BATMAN-TCM database. Previous studies have shown that they were related to the occurrence and development of bladder cancer. PPARG is a ligand-activated nuclear receptor and has been reported to interact with multiple signaling pathways, including Bcl-2, p53, p21, PTGS2, and cyclin D1 (39). The reduction of the activity of PPARG, whether through drug inhibition or gene ablation, would inhibit the proliferation of bladder cancer cells (40-42). PTGS2 is a prostaglandin endogenous peroxide synthase. Recent studies have shown that human bladder cancer is associated with increased expression of PTGS2. Compared with normal bladder tissue, the level of PTGS2 in the bladder tissue of patients with cystitis or bladder cancer is increased (43). PTGS2 is usually an indicator of poor prognosis for patients (44). Cekanova *et al.* (45) suggested that overexpression of PTGS2 could be a target for detection and treatment of bladder cancer. From our data, the mRNA expressions of PPARG and PTGS2 were up-regulated in two bladder cancer cell lines in comparison with the normal cells SV-HUC-1 (Figure 5B). Importantly, SZY-200, CP-4126, and GEM could down-regulate the expressions of the two genes in bladder cancer cells (Figure 5C). As shown in Figures S4 and S5, according to the KEGG biological pathway analyses, we found that these two genes (black arrows) are located downstream of the target genes of GEM. That’s why these drugs could reduce the expression of these two genes.

**Table 1:** List of primers for quantitative real-time PCR

**Gene**	**Primer**	**Sequences（5'→3’）**
hENT1	Forward	CCACTCAGTATTTCACAAACCG
	Reverse	ATGACATTGTTGAAGATGGCAC
PPARG	Forward	AGATCATTTACACAATGCTGGC	
	Reverse	TAAAGTCACCAAAAGGCTTTCG	
PTGS2	Forward	TGTCAAAACCGAGGTGTATGTA	
	Reverse	AACGTTCCAAAATCCCTTGAAG	
18S	Forward	CGGCTACCACATCCAAGGAAG	
	Reverse	AGCTGGAATTACCGCGGCT	

GEM: gemcitabine; EA: elaidic acid; LA: lauric acid

## Conclusion

In summary, these results indicate that SZY-200 has broad-spectrum anti-cancer activity, and the inhibitory effects on the proliferation of bladder cancer cells are comparable to GEM and CP-4126. The decrease in cell viability could be attributable to both cell-cycle arrest and apoptotic cell death. Additionally, similar to CP-4126, SZY-200 is expected to overcome GEM resistance induced by low hENT1 expression. Overall, SZY-200 has potential clinical application value. Further studies are required to determine whether SZY-200 has a favorable anti-tumor activity *in vivo* compared with GEM and CP-4126.

## Authors’ Contributions

PS and HW Conceived and designed the study; HW Analyzed data and prepared the draft manuscript; ZS, ML, and XM Critically revised the paper; ZX, BY, and QZ Supervised the research; PS, HW, ZS, ZX, BY, ML, QZ, and XM Approved the final version to be published.

## Conflicts of Interest

The authors declare there are no conflicts of interest
